# Pulmonary Hypertension: More To Be Done

**DOI:** 10.4103/1817-1737.53343

**Published:** 2009

**Authors:** Majdy M Idrees

**Affiliations:** *Head, Saudi Advisory Group for Pulmonary Hypertension and Pulmonary Medicine, Riyadh Military Hospital, Saudi Arabia*

Pulmonary hypertension (PH) is a very complex disease, characterized by a progressive increase of pulmonary vascular resistance and pulmonary artery pressure, leading to right ventricular failure and death.[1] The median survival from the time of diagnosis in untreated patients with idiopathic pulmonary arterial hypertension (IPAH) is 2.8 years.[2] In 1998 alone, there were 7,139 deaths and 174,854 hospital visits among persons with PH.

In 1987, three PH patients decided to change this very dismal picture for those who suffered from PH. At that time there was not much hope and little knowledge, secondary to very few and inconsistent research activities. The three patients, Dorothy Olson, Teresa Knazik, and Shirley Brown met each other through the National Organization for Rare Disorders (NORD) and were soon joined by a fourth person, Pat Paton. These four agreed to organize their work in order to: help patients and their families in coping with this devastating disease, provide networking opportunities to end the feeling of loneliness of a rare illness, assist in locating doctors and medical facilities with expertise in managing PH, form support groups to allow patients to communicate with others, and begin to publish a newsletter that would build a sense of community.

Only three years later, the Pulmonary Hypertension Association (PHA) was formed by more than 100 members, and a newsletter, “Pathlight”, was published. A Scientific Advisory Board was created and a PH hotline number for patients (manned by a patient volunteer) was established. In 1994, PHA held its first International Conference. Since 1994, PHA has grown to an organization of more than 3,400 members, which includes patients, caregivers, physicians, and other professionals treating the disease.

The Saudi Advisory Group for Pulmonary Hypertension (SAPH) has a very similar history. After recognition of lack of PH awareness among local physicians and caregivers, and the very poor outcome of this devastating disease that affects many patients in Saudi Arabia, five Saudi physicians constituted the nucleus of SAPH in 2004. The mission of SAPH was to devote its efforts toward the patients suffering from PH, by support, education, and treatment. The initial goals of SAPH were to carry out a national registry of PH cases all over the Kingdom of Saudi Arabia (KSA), to determine the reliable prevalence of PH in the KSA, to establish updated and complete guidelines for the diagnosis and treatment of this disease, to increase the awareness of PH, and to promote the interest for epidemiological and interventional studies in the field of PH.

By 2009, the number of SAPH members increased to more than 65, mostly physicians. The followings were achieved:

1. SAPH is now recognized regionally and somewhat internationally for its services in the field of PH. Since 2007, it has joined and represents the Eastern Mediterranean Region (EMR) office for the global Pulmonary Vascular Research Institute (PVRI)
2. Publication of Saudi Guidelines for the Diagnosis and Management of PAH[3]
3. SAPH has started a national registry for PH in the KSA
4. Research work: SAPH has recognized the importance of research, and continues to work on raising funds for this particular issue. At present, different research proposals have been forwarded to the SAPH research committee for approval, such as, prevalence of sickle cell and PH in Saudi Arabia (EMR project), Schistosoma and PH in Saudi Arabia (EMR project), high altitude PH, Saudi Experience, genetic studies in Saudi patients with congenital heart disease, Bronchiectasis and PH in Saudi patients, role of selective therapy in submassive pulmonary embolism, and lloprost experience in PH patients in a tertiary care Saudi hospital,
5. Website: SAPH has finalized the website, which has both English and Arabic locations. The website address is: www.saph.med.sa. The website describes all the activities of the different task forces and is open to the general public for communication.
6. Scientific and education: Annual PH Scientific Meetings: Two scientific meetings have already been carried out. The first and second were conducted in Dubai, UAE and Casablanca, Morocco, in the years 2007 and 2009, respectively. The Third Joint (SAPH / PVRI) International PH Meeting (and the First joint meeting with the Saudi Advisory Group for Venous Thromboembolism, SAVTE) will be held in Sharm El-Sheik, Egypt in April 2010. The scientific activities have also included awareness days for physicians and nurses, PH day, and awareness materials. SAPH is educating the community and the patients (and their families) by distributing articles and media presentations in both Arabic and English. SAPH has decided to start training sessions for Pulmonary and Cardiology Fellows in the field of PH.

We, in the SAPH, invite all interested physicians, caregivers, and patients to join this group. We hope that our effort will meet the effort of the others in order to reach our target and to fulfill our mission toward our patients.
